# ‘Women think pregnancy management means obstetric ultrasound’: Vietnamese obstetricians’ views on the use of ultrasound during pregnancy

**DOI:** 10.3402/gha.v8.28405

**Published:** 2015-10-29

**Authors:** Kristina Edvardsson, Sophie Graner, Lan Pham Thi, Annika Åhman, Rhonda Small, Ann Lalos, Ingrid Mogren

**Affiliations:** 1 Department of Clinical Sciences, Obstetrics and Gynecology, Umeå University, Umeå, Sweden; 2 Judith Lumley Centre, La Trobe University, Melbourne, Australia; 3 Department of Women's and Childrens Health, Karolinska Institute, Stockholm, Sweden; 4 Department of Medicine, Centre for Pharmacoepidemiology, Karolinska Institute, Stockholm, Sweden; 5 Department of Dermatology and Venereology, Hanoi Medical University, Hanoi, Vietnam

**Keywords:** maternal rights, sex selection, obstetric ultrasound, obstetricians, pregnancy, pregnant women, prenatal diagnosis, qualitative studies, Vietnam

## Abstract

**Objective:**

To explore Vietnamese obstetricians’ experiences and views on the role of obstetric ultrasound in clinical management of complicated pregnancy and in situations where maternal and fetal health interests conflict.

**Design:**

Seventeen obstetricians in northern Vietnam were interviewed as part of the CROss-Country Ultrasound Study (CROCUS) project in 2013. Data were analysed using qualitative content analysis.

**Results:**

The participants described ultrasound as a central tool in prenatal care, although they called for increased training and resources to prevent inappropriate management. A prevailing overuse driven by women's request and increased commercialisation was described. Other clinical examinations were seen as being disregarded by women in favour of ultrasound, resulting in missed opportunities for identifying potential pregnancy complications. The use of ultrasound for sex selection purposes raised concern among participants. Visualisation of human features or heartbeat during ultrasound was commonly described as the point where the fetus became regarded as a ‘person’. Women were said to prioritise fetal health interests over their own health, particularly if a woman had difficulties becoming pregnant or had undergone assisted fertilisation. The woman's husband and his family were described as having an important role in decision-making in situations of maternal and fetal health conflicts.

**Conclusions:**

This study provides insight into issues surrounding ultrasound use in contemporary Vietnam, some of which may be specific to this low-income context. It is clear that ultrasound has become a central tool in prenatal care in Vietnam and that it has also been embraced by women. However, there seems to be a need to balance women's demands for obstetric ultrasound with better recognition of the valuable contribution to be made by the full range of clinical examinations in pregnancy, along with a more strategic allocation of resources, that is, use of obstetric ultrasound based on clinical indications. Better regulation of private obstetric practice also appears to be needed. While the root causes of sex selection need to be addressed at societal level, efforts are also required more immediately to find ways to combat the inappropriate use of ultrasound for the purpose of sex selection.

The use of ultrasound in pregnancy is routine practice nowadays in the developed world and is also rapidly becoming routine practice in resource-poor settings (1, 2). The benefits of ultrasound as a diagnostic tool in high-risk pregnancies in both high-income and low-income settings is largely undisputed (3, 4), and the routine use of ultrasound has shown to incur benefits, including assessment of gestational age, detection of multiple pregnancies (5), and the detection of fetal anomalies (6). However, there is still a debate about whether the routine use of ultrasound during pregnancy brings benefits to the mother and baby when used in unselected or low-risk populations (1, 5). In Vietnam, obstetric ultrasound is now considered an indispensable part of antenatal care (ANC), and almost all Vietnamese women undergo ultrasound during pregnancy (7, 8). Pregnant women in Vietnam are advised to attend ANC in each trimester, with more frequent examinations (once a month) in the last trimester. Ultrasound is recommended to be part of the assessments at 11–13, 18–22, and 28–32 weeks (9). However, it is common for women to have more frequent scans, with the average number of scans reaching six in urban areas and 3.5 in rural areas (7, 8).

Many studies from different parts of the world have confirmed the popularity of ultrasound among pregnant women and their partners, and besides reassurance, reasons for undergoing a scan also include factors such as ‘meeting the baby’ and visual confirmation of the reality of the pregnancy (10). Ultrasound has been shown to be important to pregnant women in Vietnam as well, and wanting reassurance of normal fetal development has been described as one key reason for having frequent scans (7). Ultrasound is also valued by health care providers, especially those who work as private providers, perhaps because it can provide significant revenue in the increasingly commercialised Vietnamese health care system. It is common for doctors to run their own private practice outside their working hours in the public system in order to increase their income (7). Furthermore, the Vietnamese government has limited influence and control over the health care provided in the private sector (11), including the provision of ultrasound in pregnancy.

Although the benefits of diagnostic ultrasound in resource-poor settings have been established (4), significant drawbacks have also been acknowledged. Vietnam is characterised by a patrilineal and patrilocal kinship system, which gives rise to a deep-rooted preference for sons (12). This in combination with increasing availability of sex selection technology, including ultrasound (12), has led to a rising sex ratio at birth (SRB, the number of boys born per 100 girls) in the country during the past decades (13–16). Thus, the practice of sex determination for selective abortion of female fetuses is a recognised problem, despite the introduction of a policy in 2003 that prohibits both sex determination and sex selection (17). Other identified drawbacks of the use of ultrasound in resource-poor settings include overestimation of diagnostic capacities and therapeutic possibilities by both providers and pregnant women, and as previously mentioned, overuse because of financial incentives for health care providers (4).

Although almost all pregnant women in Vietnam nowadays attend some form of ANC (8), there are big disparities in ANC utilisation between rural and urban areas, and large inequities along socio-economic and ethnic lines (18). For example, only 20% of pregnant women in rural areas receive all core ANC services with a reported average of 4.4 visits, while the figure for their equals in urban areas is 81% with an average of 7.7 visits (8). Furthermore, the ANC that Vietnamese women receive does not always fulfil the national recommendations, especially in the private sector, and the adequacy of ANC use has been shown to be poor, particularly among women with low education, low economic status, and those living in rural areas (19).

To date, there is a lack of studies investigating the implications of the use of ultrasound in pregnancy in low-income settings from the perspective of the health care providers. In this study, we aimed to explore Vietnamese obstetricians’ experiences and views on the role of obstetric ultrasound in relation to clinical management of complicated pregnancy and in situations where maternal and fetal health interests conflict.

## Methods

### Study design

The study, undertaken as part of the CROss-Country Ultrasound Study (CROCUS) project (20), had a qualitative design involving semi-structured interviews with medical doctors working in prenatal care in northern Vietnam.

### Participant recruitment

We aimed for diversity in relation to demographic characteristics of participants, as well as in relation to the level of health care in which they were practising. Purposive sampling was therefore applied and participants were recruited from six different district-level, city-level, and national-level hospitals in northern Vietnam. Two of these were private hospitals. The characteristics of the included hospitals are presented in Table 1. One member of the research group (LPT), who is a dermato-venereologist practising in Hanoi, Vietnam, initiated all contacts and approached colleagues/directors of each district- and city-level hospital for approval to recruit participants. A coordinator or staff member appointed by each hospital director assisted in recruitment, and LPT visited all hospitals to provide information on criteria for recruitment. The doctors at the national-level hospitals were contacted directly by LPT who had relevant personal and health system knowledge (the interviews were subsequently performed by SG and IM). The participants were offered remuneration of US$20 for their participation following the interview but did not know beforehand that they would get paid.

**Table 1:** Key characteristics of the six hospitals located in Northern Vietnam

Hospital	No. of participants	Level of health care	Geographic area	Intensive care available	NICU^a^ available	Obstetric ultrasound performed by:
1	4	District level	Rural	No	No	Sonographers^b^ Obstetricians
2	4	City level	Suburban	Yes	Yes	Sonographers^b^ Obstetricians
3	3	City level	Urban	Yes	Yes	Sonographers^b^ Obstetricians
4	1	City level	Urban	NA	NA	Sonographers
5	4	National level	Urban	Yes	Yes	Obstetricians
6	1	National level	Urban	Yes	Yes	Obstetricians

a Neonatal Intensive Care Unit.

b Medical doctors mainly responsible for obstetric ultrasound examinations.

### Participants

Inclusion criteria for participation were being a doctor working with obstetric ultrasound examinations as a major work task, doing obstetric ultrasound examinations as part of general obstetric care, or using the results of obstetric ultrasound in clinical management of pregnant women. In total, 17 participants were recruited, nine males and eight females. The mean age was 45 years (range 28–54 years). They were all medical doctors; 14 had specialised in obstetrics and gynaecology, and three in sonography. Two of the participants were heads of department of obstetrics and gynaecology at their hospitals.

### Data collection procedures

The data collection was performed over 2 weeks in March 2013 by SG, LPT, and IM. The approximate number of 12–15 participants was planned before the start of the data collection given our experiences in previous interviews in Sweden and Australia. An interview guide was developed as part of the CROCUS study (Table 2), and it was used to ensure that all topics of interest were covered. The topics were not raised in a predefined order, which meant that the interviewer could follow topical trajectories and by probing encourage the participant to express their experiences and views at length. All interviews were held at the hospitals in separate rooms to avoid interruptions. One interview was held in English and 16 were held in Vietnamese with the assistance of an interpreter (LPT or LVH). The interviews lasted between 35 and 63 min (mean 48 min) and were recorded digitally with approval from the participants. After each interview the interviewers did a brief joint analysis of the content and data collection continued until saturation was obtained after 17 interviews, with no new data emerging.

**Table 2:** Key domains in the CROCUS interview guide

Key Domains
The obstetricians’ experiences and views of: • the importance/value of obstetric ultrasound for clinical management of complicated pregnancy, • the importance of obstetric ultrasound in comparison to other surveillance methods during complicated pregnancy, • clinical situations where the interests of maternal and fetal health conflict, • whether the woman may be considered to act as an instrument for fetal treatment, • if/when the fetus can be regarded as a person, • situations where the fetus has been regarded as a patient with his/her own interests, • their professional role in relation to other occupational groups working with obstetric ultrasound examinations or the outcomes of these examinations, and • other issues in relation to ethical aspects of the use of obstetric ultrasound.

### Data analysis

The recorded interviews were transcribed verbatim and then translated into English by a member of the research group (LVH). KE, IM, and LPT read all interviews several times to get a sense of the whole. KE coded all data and sorted the codes into broad content areas based on similarities and differences, and then further refined the data and labelled categories and sub-categories. KE, IM, and LPT then met to discuss the emerging results and the interpretations made. These discussions resulted in further refinement of the content and labelling of categories. All authors were then given an opportunity to comment on the developing results. Discrepancies in interpretations were resolved through discussions between all authors until consensus was obtained.

### Ethical considerations

Written invitations were sent to the potential participants and verbal informed consent was obtained from all participants at the time of each interview. All participation was voluntary. Ethics approval was obtained from Hanoi Medical University review board (HMUIRB) in Bio-Medical research (reference 141/HMU IRB).

### The researchers’ backgrounds

The research group represents both qualitative and quantitative research traditions and various professional disciplines, including midwifery, nursing, maternity services and maternal health research, obstetrics and gynaecology, venereology, behavioural science, public health, and epidemiology.

## Results

The analysis resulted in three main categories that each included two-to-three sub-categories, which describe the participants’ experiences and views on the role of obstetric ultrasound in relation to clinical management and situations where maternal and fetal health interests conflict (Table 3). Each category is presented below together with illustrative quotations from participants (italics).

**Table 3:** Categories and sub-categories of obstetric ultrasound use in Vietnam

Integrating new imaging techniques in a low-income setting	Ultrasound – a central tool in pregnancy management
	Community misconception about ultrasound's role in pregnancy
	Commercialisation of ultrasound
Experiencing changing perceptions and actions due to visualisation of the fetus	The role of ultrasound in defining the fetus as a person Dealing with community preference for male fetuses
Managing situations of maternal and fetal health conflicts	The professional challenge of caring for two ‘patients’
	Facing women's self-sacrifice
	Pregnancy management a family issue

### Integrating new imaging techniques in a low-income setting

#### Ultrasound – a central tool in pregnancy management

The participants regarded ultrasound as a central and important tool in obstetric practice, something most obstetricians felt they required for adequate surveillance of the fetus, particularly in complicated pregnancy.

Ultrasound is a big step for specialised obstetrics. The obstetric ultrasound room can be considered as the heart of the obstetric department. (No. 17)

It was described largely as a method for monitoring the status of the fetus, with the advantage of helping doctors to make the right clinical decisions about intervening in time. While some of the participants did not perform ultrasound examinations themselves but used the results of ultrasound in pregnancy management, others performed large numbers of examinations each day. One participant said the number of ultrasound scans performed by one operator per day could be as many as 100, or even higher depending on the level of the hospital.

Although the participants described ultrasound as the most commonly used investigation during pregnancy, they emphasised the importance of combining ultrasound use with other clinical examinations and tests for proper pregnancy management. Every investigation was said to have its advantages and disadvantages. However, ultrasound during pregnancy was seen as very advantageous due to its noninvasiveness and simplicity (to perform), while at the same time providing direct and comprehensive information.

I think that in Vietnam nowadays, obstetric ultrasound is the most important investigation to monitor the pregnancy. Some other investigations like blood test, urine test also have importance but they cannot be compared to the obstetric ultrasound. (No. 17)

Furthermore, the participants perceived ultrasound to be safe to use without risk to the pregnant mother and the fetus and none raised concerns over possible physical side effects.

So far, I have never known any report about the harm of the ultrasound to the mother and the fetus. In our hospital we view ultrasound as indicated for almost 100% of cases because of its high value and safety. (No. 2)

Some of the participants described themselves as highly qualified and experienced in performing obstetric ultrasound, while others were less confident using the technique because of lack of training. Some of the participants raised concerns over the fact that many doctors who performed ultrasound examinations lacked proper training, something they believed sometimes led to inappropriate management.

I have seen our colleagues practise incorrectly many times because they don't have enough training. (No. 1)

#### Community misconception about ultrasound's role in pregnancy

It was a shared experience among the participants that obstetric ultrasound was seen as a very important examination in the community. They stated that pregnant women in general requested repeated ultrasounds, and that women who were not routinely offered ultrasounds (without medical indication) when visiting the clinic became dissatisfied. Common reasons for wanting many ultrasounds were described as including ‘seeing the baby’ and visually following the development of the fetus.

Vietnamese [pregnant women] like ultrasound [during pregnancy] very much so any time they visit the hospital they want to have an ultrasound. (No. 9)

Many participants perceived ultrasound to be the examination pregnant women had the highest confidence in, and they stated that it was common for women to come to the hospital only for the ultrasound, without undergoing other medical examinations related to pregnancy management. The participants thought that by having an ultrasound examination, pregnant women commonly believed they had had ‘a complete check’ of the pregnancy, and clinical examinations and other tests were therefore perceived as redundant. This could sometimes result in potential pregnancy complications, such as high blood pressure and developing pre-eclampsia not being picked up by doctors.

In Vietnam, most of the pregnant women have mistaken their thinking about pregnancy management. They consider that pregnancy management means obstetric ultrasound. That is the reason why they only go to have an obstetric ultrasound and they think that it is enough. It is lucky for them if the people who do the ultrasound are obstetricians. But it is not good if they are not obstetricians. (No. 17)

I have asked many patients here ‘Have you ever had the examination before?’. They answer, ‘Yes, I have already visited a doctor many times’. But when I ask them to give me the medical record, there are only the results of ultrasound [examinations] there. (No. 10)

Some participants thought that people in the community had poor knowledge about pregnancy-related health problems and fetal abnormalities, and therefore did not worry much about such issues. It was emphasised though that people with high levels of education also had a better understanding of ultrasound and pregnancy complications. It was suggested that they therefore would discuss the situation with the doctor to a greater extent, in contrast to those with low education who would rely more often on the doctor's decision. Some participants also raised the issue that doctors who performed ultrasound were usually very busy and, therefore, did not take time to explain and discuss the results of the ultrasound with the pregnant women. According to the participants, this meant that pregnant women who lacked medical knowledge would usually not understand the information yielded from the examination. One participant implied that colleagues were sometimes blamed if a baby was born with a disease or fetal malformation.

#### Commercialisation of ultrasound

The participants reflected on the current overuse of obstetric ultrasound, that is, that most women underwent more ultrasound examinations than was medically indicated. Some claimed that ultrasound was increasingly being commercialised. However, while some doctors said they tried to inform women that there were no indications for additional ultrasounds, others said that women's requests were often met, particularly by doctors who had an economic incentive for performing many scans.

In some areas doctors spend a lot of money buying 3-D, 4-D ultrasound machine (about 16000–17000 USD) so they want to get it back quickly. For them, it is easy [to perform many ultrasound examinations] because some families want to do that too. (No. 1)

I tell you the truth that almost all obstetricians like ultrasound very much because it is easy to operate and easy to get money. (No. 16)

### Experiencing changing perceptions and actions due to visualisation of the fetus

#### The role of ultrasound in defining the fetus as a person

The participants had different views about when the fetus could be regarded as a ‘person’. The majority described the moment when the heartbeat, human features, or body movements could be visualised through ultrasound as the moment when the fetus became a person. Thus, the obstetric ultrasound was clearly described as contributing to ‘personification’ of the fetus.

I consider the fetus as a person when we identify the heart beat under ultrasound. (No. 12)

I consider a fetus as a person when I can see some part of the body like the hand, foot, heart, face … under ultrasound. (No. 8)

Some participants presented the view that the fetus becomes a person even at an earlier stage, from conception or from the stage when a woman would recognise the symptoms of pregnancy or show signs of pregnancy such as a late menstruation or positive pregnancy test. In addition, a few stated that the fetus did not become a person until it was viable outside the womb. Some participants suggested that in the past, when there was no ultrasound and when the fetus could not be visualised, it was easier for ‘families’^1^ as well as doctors to decide to terminate a pregnancy if there were complications or if the mother was too ill to continue the pregnancy. However, seeing the heartbeats, the face of the fetus, or the fetal movements was said to bring the fetus emotionally closer to both the doctor and the family, making decisions about termination even more difficult for both parties.

When the parents and their family can clearly see the image of the baby, they feel love for the baby much more and the relation between the mother, the family and the fetus becomes closer. They consider the fetus as a real person. (No. 2)

With doctors, their emotion in relation to the fetus also changes a lot when they can see the image of the fetus under ultrasound. (No. 1)

#### Dealing with community preference for male fetuses

A recurrent issue mentioned by participants was the prevailing preference for male fetuses in Vietnam, and the significance of ultrasound for the possibility of selective abortion was raised in some interviews.

If you would like to understand about the situation of obstetric ultrasound in Vietnam you should raise the questions of sex preference …. Many people don't want to have a baby girl for two reasons. Firstly they think that the life of a girl is not as good as a boy. The second reason is that if the woman gives birth to a boy, her mother-in-law will be very happy. (No. 9)

According to the participants, doctors were not allowed to report the sex of the fetus, and they emphasised that they would never reveal the sex of the fetus to pregnant women who they thought would use the information for deciding on sex-selective abortion. Some said they would only reveal the sex of the fetus to close friends who they trusted would give birth to the baby regardless of the sex. However, several stated that expectant parents commonly knew the sex of the fetus because of the accessibility of private ultrasound providers.

The regulation from the Ministry of Health, even they forbid telling the sex …. Only in the public hospital do they follow that rule. However, in the private hospital and clinic they normally report the sex. So if we forbid in the hospital, the parent will go to the private clinic to do more ultrasound. (No. 9)

By having obstetric ultrasound we can easy know the sex of the fetus, and based on the result of ultrasound many people may go for induced abortion. This makes me very upset and feel pain, and I am against this. (No. 11)

The prevailing preference for male offspring was also illustrated in one of the participant's claims that women would look after themselves more if they carried a male fetus; some women who knew that the fetus was a girl cared less about the situation of the fetus.

### Managing situations of maternal and fetal health conflicts

#### The professional challenge of caring for two ‘patients’

The participants gave several examples of situations where the health interests of the pregnant woman and the fetus had been in conflict, the most common being pregnant women who had developed pre-eclampsia at a stage when the fetus was not mature enough for extra-uterine life. Other situations included heart, kidney, or liver problems in pregnant women, as well as other conditions where the woman's health deteriorated greatly because of the pregnancy. Participants working at the low-level hospitals described being less commonly exposed to conflicting situations than their colleagues in the higher-level hospitals because complicated cases were referred to the latter for clinical management. The majority of participants said that the health of the woman was always prioritised; however, some ambivalence was discerned in relation to this question as the circumstances in each case were said to influence decision-making. For example, the interests of the fetus were said to be given more weight in cases of infertility and assisted fertilisation.

It depends on the gestational age and the disease of the mother which person is most important (mother or fetus). (No. 3)

Balancing of maternal and fetal health interests depends on each case. Such as in the case [where] the mother already has children, if the treatment for the fetus harms the mother, normally we give priority to the mother. In contrast, in the case of infertility, normally the family will try their best to give priority to the fetus. (No. 10)

I think that [in] all of the cases we try to protect the mother more than the fetus. Even [when] we clearly see the fetus under ultrasound. (No. 8)

One participant described a couple of cases where the health interest of the fetus had been given priority over the health interest of the mother. However, these situations were considered exceptional and were characterised by serious maternal conditions that were not possible to treat, and maternal death would inevitably occur. The participants indicated that decisions in situations of maternal–fetal conflicts were hard to make, and such situations were described as very stressful for the obstetricians.

### Facing women's self-sacrifice

The participants unanimously stated that women would always agree to treatment for the benefit of the fetus or to save the fetus. It was described as extremely rare that a woman would not follow the doctor's advice if it was in favour of the fetus.

I think naturally, all pregnant women always expect to have a good outcome with a healthy baby, therefore, whenever their fetus is having some problems, the pregnant women will try their best and agree to get treatment for the baby. (No. 7)

Some participants felt that the issue of women refusing interventions for the benefit of the fetus was more of a problem in developed countries rather than in Vietnam, as women in more developed countries were more knowledgeable about their own potential health risks. While some participants thought that there were many ethical aspects involved when the interests of maternal and fetal health were not aligned, others saw no ethical problem in these situations:

I think there is no ethical problem because every pregnant mother will sacrifice their best thing to protect their baby. (No. 3)

In Vietnam, most of the woman will follow the advice of the doctor to save the baby. (No. 4)

However, the situation was described to be different if the doctor advised mothers on termination if her own health or life was at severe risk because of the pregnancy. In such circumstances, it was said that women often tried to continue the pregnancy despite the risks to her own health, particularly if the woman had difficulties getting pregnant or if she had undergone assisted fertilisation. Having to terminate a pregnancy because of risks to the pregnant woman's own health was described as a very stressful situation for the woman as well as for her partner or family.

Couples who did not get pregnant easily try to protect the fetus even when they understand the risk for the mother. (No. 8)

#### Pregnancy management a family issue

Situations of maternal and fetal health conflicts were said not only to be a matter of concern to the pregnant woman and the doctor but also described as a family issue in the Vietnamese setting as the pregnant woman's husband and his family have an important role in decision-making. Sometimes, family members were said to worry a lot over the woman's health and therefore asked the doctor to terminate the pregnancy to save the mother's life. On the contrary, sometimes members of the family did not understand the severity of the health risk to the pregnant woman and they therefore wanted to prioritise the baby.

The 2nd example [of when the fetus is considered more] is in the case where the mother has cardiac disease. In that case the fetus raises the risk to the mother, but their family sometimes still would like to try to protect the fetus inside the mother [as long as possible]. (No. 5)

The doctors described how they tried to provide information so that the woman and the family could make an informed decision in situations of maternal–fetal health conflicts. Moreover, the woman and family were said ultimately to follow the doctor's advice, thus decisions were described as not being entirely in the hands of the pregnant woman. Sometimes the knowledge level of the family was low, which made it challenging for doctors to explain what was happening to ensure the family understood what was going on with a complicated pregnancy and the risks. However, for the woman and the family to make a decision on management, also other aspects were said to come into play, such as the cost for medical treatment, hospitalisation, food, et cetera.

We should explain to them [the family] and try to give them two possibilities: the first is the percentage of safety if we keep the baby or keep the pregnancy going on, what percentage that the mother dies, and what percentage to save the life of the fetus. And then the family can discuss with us about what they want. (No. 1)

For example in the case of pre-exclampsia, we try to protect the mother but sometimes we cannot and it can affect the baby. And mostly in that case we will try to protect the mother, but we still discuss with the family to make them understand clearly and together with us to make a suitable decision.

## Discussion

The overall purpose of this study was to explore Vietnamese obstetricians’ experiences and views on the role of obstetric ultrasound in clinical management of complicated pregnancy, and in situations where maternal and fetal health interests conflict. The results outlined three main categories that provide insight into the issues surrounding the use of ultrasound, many which may be specific to Vietnam's context as a low-income setting.

First, the category ‘Integrating new imaging techniques in a low-income setting’ clearly depicts the current overuse of ultrasound in Vietnamese prenatal care. The two key drivers for overuse of ultrasound as identified in this study seem consistent with findings from a previous study from Vietnam: women's desire for reassurance of normal fetal development and the growing commercialisation in the Vietnamese health care system, which provides opportunities for providers or health facilities to obtain significant revenue from providing ultrasound examinations, especially in private settings (7). There are several implications of overuse of obstetric ultrasound. One is that it undeniably leads to increasing patient loads, and therefore, decreasing quality of care and skewed allocation of scarce resources (7, 21). Also, there are strong reasons to believe that a doctor who performs a large number of scans per day does not have time for proper information giving and counselling, which means that pregnant women risk getting insufficient information about the indication, benefits, process, limitations, as well as the results of the scan. It also means that the process of informed consent is undermined. These issues deriving from limited doctor–patient contact have been identified in previous studies from Vietnam as well as in other low-income settings (7, 21, 22). Another issue relates to safety, as there are still uncertainties around what limits of exposure of energy related to ultrasound can be considered safe in pregnancy. The World Federation for Ultrasound in Medicine and Biology recommends prudent use of ultrasound in pregnancy and disapproves nonmedical use because there is still not enough evidence of safety for the embryo or fetus (23). Thus, there seems to be a clear gap between these guidelines and the situation in relation to the use of ultrasound in Vietnam. Possible negative effects of the multiple scanning that takes place in Vietnam as well as in some other similar settings (24, 25) are yet to be elucidated.

Another important finding of this study is that women may disregard clinical examinations in favour of ultrasound because the ultrasound may be perceived as a ‘complete check’ of the pregnancy. It is possible that this is caused by a number of factors, including the huge popularity of ultrasound among pregnant women as well as health care providers, an overestimation of the benefits of ultrasound, and a lack of continuity of care for pregnant women in Vietnam. The implications for maternal and fetal health outcomes are important to consider. Pregnancy complications that cannot be revealed by an ultrasound investigation (high blood pressure, gestational diabetes, pre-eclampsia, etc.) that may develop during the course of pregnancy risk being overlooked, which means that some women will not get appropriate and timely treatment to optimise maternal and fetal health. Our study results confirm findings of a previous Vietnamese study that identified a tendency for women to replace ANC visits with ultrasound scanning: the women found ultrasound superior to other examinations and some felt that they did not need ANC as it provided them with very little additional information (7). This phenomenon has also been identified in an African low-income setting among both health care providers and pregnant women, that is, the risk of de-valuing nontechnological procedures such as history taking and physical examination in pregnancy in favour of the use of ultrasound (21). Thus, this growing knowledge provides evidence for the need to reinforce the role of clinical examinations and balance women's demands for ultrasound with the known contribution that clinical examinations make.

The second category ‘Experiencing changing perceptions and actions due to visualisation of the fetus’ brings up the issue of the existing preference for male fetuses in Vietnam and the significance of ultrasound for the possibility of sex-selective abortion. According to the World Health Organization, in some parts of the world an accelerated increase in the sex-ratio imbalance at birth has been seen since ultrasound and other diagnostic technologies became available in the early 1980s (26). There are indications that sex-ratio imbalance has grown in many South Asian, Central Asian, and East Asian countries in the recent decades as a result of preference for the male child and sex selection (26). A trend towards increasing imbalance in SRB (13) from the biological value of 105 has been documented in Vietnam since the year 2000 (13–15), reaching 110.6 at the 2009 census (13). An elevated SRB was also demonstrated in the Quang Ninh province in northern Vietnam during the years 2008–2011, with large differences in SRB depending on both place and mode of delivery (16).

Although there are clear signs that sex-selective abortion following prenatal sex determination is an increasing problem in Vietnam (14), it is important to note that the use of ultrasound or other sex selection techniques at different stages of reproduction are not causative factors for sex selection (26–28). Biased sex selection has its origin in gender-based discrimination, and these techniques are thus only a means to achieve this. Some Asian countries, including Vietnam, have implemented laws and policies relating to sex selection and abortion (27); however, it is argued that these legal measures may have limited effect if they are implemented without efforts aimed at changing social norms and structures (26, 27). This resonates with our study results indicating that expectant parents commonly knew the sex of the fetus and that the practice of sex-selective abortions was an existing phenomenon despite the fact that a policy prohibiting both sex determination and sex selective abortion has been in place since 2003 (17). Thus, it is important for governments to address the root cause of gender-biased sex selection, that is, the underlying social and gender inequalities (26) in addition to legislating against sex-selective abortions that have been inadvertently facilitated in recent decades by the ease of fetal sex identification by ultrasound.

The third category ‘Managing situations of maternal and fetal health conflicts’ illustrates how situations with clashing interests of maternal and fetal health can create difficulties for the woman herself, the health professionals, and the family. Women were said to prioritise fetal health interests over their own health, particularly if a woman had difficulties getting pregnant or if she had undergone assisted fertilisation. These situations sometimes seemed supported by both doctors and families, something that has to be understood in the context of the significance of childbearing in Vietnam. For a woman, having children is important to fulfil a maternal identity, to create a good relationship with the husband, to continue the family line, particularly for women married to the oldest son in a family, for providing security for the future, and also to achieve a positive status in the husband's family. Having children has also been described as an important factor for overall happiness and harmonious social relationships within the family (29, 30). Women who fail to produce offspring, particularly male offspring, may experience social and psychological suffering, the risk of being rejected by the husband or his family, and may even be exposed to conflicts or domestic violence (12, 31).

The finding that the family has an important role in pregnancy-related decision-making is consistent with previous research (32). The existing patrilineal and patrilocal kinship system that exists in Vietnam may significantly interfere with women's reproductive freedom. Women, who after marriage commonly live in the household of their husband's parents, may be put under considerable pressure to produce a son, thus allowing for the family line to continue. So it is not an uncommon phenomenon that women continue to carry pregnancies until a son is born (12), which is also confirmed by the results of this study. Considering the preference for male offspring, it seems reasonable to believe that women would more commonly risk their own health to optimise fetal health in the case of a male fetus. Previous research from Vietnam has shown that sex of the fetus can influence both mode and place of delivery; mothers of boys are more likely to receive more qualified delivery care and caesarean sections (16), a finding that could be seen as supporting our hypothesis. This is an area for further study.

It is evident from this study that ultrasound has a central role in prenatal care in Vietnam. However, better regulation of private medical practice seems necessary to prevent overuse and misuse of obstetric ultrasound, as well as stronger public health messages about the need for a medical indication for medical procedures. It is also clear from the present study and previous studies that pregnancy and childbirth is a family matter in Vietnam. In efforts to reinforce the core components of ANC and the role of clinical examinations, and balance women's demands for ultrasound with the known contribution of clinical examinations, this needs to be taken into consideration.

### Strengths and limitations

A strength of this study is that participants were recruited from district-level, city-level, and national-level hospitals located in both urban and rural areas. It is likely that this contributed to a wide range of experiences and views being obtained. Limitations of the study were that the interviews needed to be performed with the assistance of an interpreter. This may have negatively influenced the use of follow-up questions and probing, as well as impeding the opportunity for participants to express their views at length.

## Conclusions

This study provides insight into issues surrounding ultrasound use in contemporary Vietnam, some of which may be specific to this low-income context. It is clear that ultrasound has become a central tool in prenatal care in Vietnam and that it has also been embraced by women. However, there seems to be a need to balance women's demands for obstetric ultrasound with better recognition of the valuable contribution to be made by the full range of clinical examinations in pregnancy, along with a more strategic allocation of resources, that is, use of obstetric ultrasound based on clinical indications. Better regulation of private obstetric practice also appears to be needed. While the root causes of sex selection need to be addressed at the societal level, immediate efforts are also required to find ways to combat the inappropriate use of ultrasound for the purpose of sex selection.

## Footnotes

1 The ‘family’ can in the Vietnamese context also include parents and parents-in-law or siblings. However, it is common that the woman's husband and his family are more involved in decision-making.
